# Medical Education in a Post COVID-19 era – remote teaching methods for cardiovascular knowledge and skills

**DOI:** 10.15694/mep.2021.000062.1

**Published:** 2021-03-03

**Authors:** Bernadeta Bridgwood, Cezar Sabbagh, John Houghton, Andrew Nickinson, Coral Pepper, Rob Sayers

**Affiliations:** 1 University of Leicester; 2 University Hospitals of Leicester

**Keywords:** Cardiovascular, undergraduate, remote teaching, curriculum planning

## Abstract

This article was migrated. The article was marked as recommended.

Introduction

As traditional clinical teaching faces major obstacles during the COVID-19 pandemic, medical educators look toward remote teaching methods to provide solutions to allow continuation of teaching. Remote methods, teaching delivered other than face-to-face, align with the transformation seen within pedagogy over the last 20 years.

Aim

The aim of this scoping review was to i) identify existing teaching methods available to remotely teach cardiovascular knowledge or skills and ii) identify if they have been evaluated.

Methods

A scoping review of the literature was undertaken to synthesise available evidence and examine remote teaching methods for application to undergraduate medical education.

Results

Forty-two articles were identified which presented remote teaching methods using either teaching based online, computer-programs, digital resources, mobile-phone technology, podcasts, serious gaming, social media or resources to aid self-directed learning. Although results were heterogenous, they gave an indication of the method’s usefulness. However, evaluations were not consistent and if they were, would have strengthened the value of the findings.

Conclusion

Various remote teaching methods are available to replace face-to-face cardiovascular teaching where this is not possible. Evidence for effectiveness and engagement of individual platforms are variable. Given the ongoing COVID-19 pandemic, medical educators should prioritise ongoing evaluation of remote teaching methods and share best practice.

## Introduction

During the ongoing worldwide pandemic of COVID-19, traditional clinical teaching faces major obstacles. Medical students are frequently unable to attend university or clinical placements. A major challenge for medical educators is to adapt to using remote teaching methods, defined as teaching delivered by means other than face-to-face, while replicating the experience of clinical encounters ( Goh and Sanders, 2020).

For over a decade, medical schools have been transforming pedagogy, moving from didactic lectures to increasing use of technology and self-directed learning (SDL) ( Shochelak and Stack, 2017). The pandemic has accelerated this process, where the rapidity of transition to remote learning has moved much faster than current innovation and identification of the evidence for different teaching methods. With such major pedagogical changes, monitoring and evaluation is integral to ensuring quality, improvement and meeting learner needs ( Jayawickramarajah, 2001).

Within this scoping review, we focus on the cardiovascular system as a broad category, including both knowledge and skill-based learning. Cardiovascular disease (CVD) is the main cause of mortality worldwide and thus, appropriate acquisition of knowledge and skills are essential for practising clinicians ( World Health Organisation, 2020). Face-to-face teaching has traditionally been important in CVD and alternative teaching methods have had to be sought during the pandemic. During the ongoing pandemic, there is an opportunity and necessity to evaluate whether alternative remote learning methods for medical education sufficiently meet student and curriculum needs. We utilised a methodological framework to scope the literature.

This scoping review aims to search the available literature to identify teaching methods which address cardiovascular knowledge, skills or both and inform undergraduate medical educators of the evidence for remote teaching methods.

## Method

This scoping review followed a 5-stage framework for scoping review proposed by Arksey and O’Malley presented within the Joanna Briggs Institute review methods manual ( Arksey and O’Malley, 2005; Peters *et al.*, 2020). In keeping with scoping review guidance, gaps in the evidence base were identified including topics for future research and methodological quality was not formally assessed.

### Planned approach

A study protocol was published on The Open Science Framework on the 7th July 2020 and can be accessed at https://osf.io/9vxd6/

### Identification of a research question

This review was guided by the research question - what are the existing methods available to remotely teach cardiovascular knowledge or skills that could be employed in undergraduate teaching and have they been evaluated?

### Identifying relevant articles

Literature searches were conducted in MEDLINE, CINAHL, Embase, ERIC from 2000 until present (5 ^th^ May 2020, updated on 15 ^th^ July 2020) using a search strategy (Supplementary File 1). This time point was chosen as medical school curriculum has evolved greatly since 2000. Additional grey literature was identified by targeting academic website domains using Google Search. Results were supplemented by scanning the references of relevant articles.

### Study Selection

Article relevance was judged by the following criteria 1) teaching methods aimed to improve cardiovascular skills or knowledge 2) partially/fully remote methods 3) article in English 4) full text articles. All publication types were included, from any location worldwide to ensure a full scope of available teaching methods. Two reviewers performed study selection and data abstraction independently. Discrepancies were resolved through discussion.

### Data charting

All records were managed using EndNote (version X9) bibliographic software. The data collection form for intervention reviews of non-randomised studies presented by the Cochrane collaboration was adapted for use within this review ( The Cochrane Collaboration, 2021). This captured publication data, setting, method used, method delivery and any evaluation of the method.

### Collating, summarising and reporting the results

Review findings were tabulated and grouped into teaching method categories. The Kirkpatrick model of Training Evaluation was used as a framework for describing the learning outcomes and evaluation in each study. Informed by our research questions, we adopted a narrative approach to summarise and report the data to provide insight regarding the content of each teaching method.

## Results

### Results

**Figure 1.**
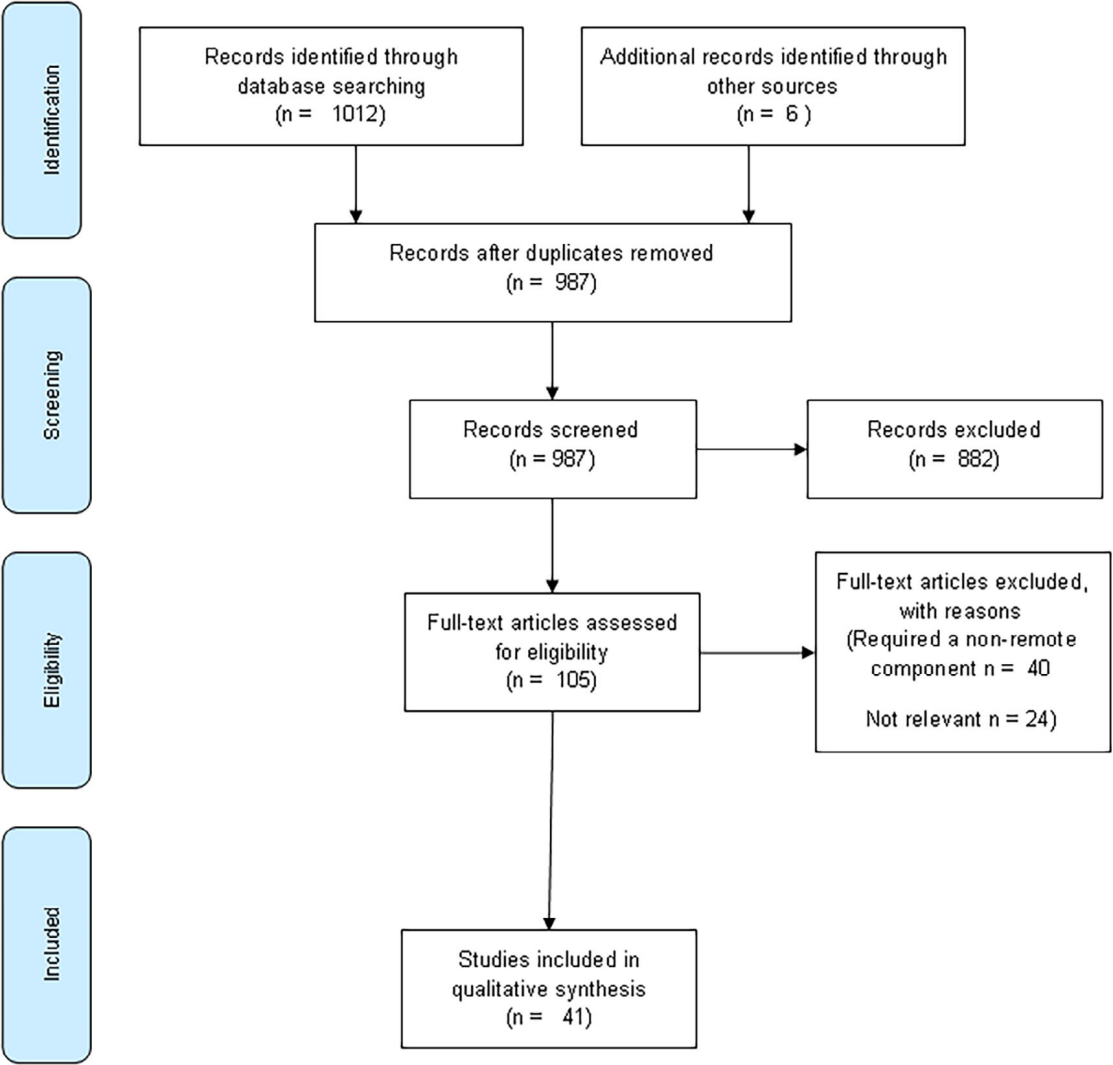
Schematic presentation of studies selected in the review

Of 1018 articles identified, 41 articles were included (PRISMA) ( Figure 1). The PRIMSA diagram details our search and selection process applied during the analysis. A summary of the results is presented in Table 1. There were eight methods of teaching delivery which included online teaching; digital resources; computer programmes; mobile learning; serious gaming; podcasts; social media and resources assisted self-directed learning (SDL).

Adaption of PRISMA model - (Moher, Liberati, Tetzlaff and Altman for the PRISMA Group, 2009)

**Table 1.** Summary of results

Author	Topic	Method	Supplement teaching	Kirkpatrick level	Educational theory
*Online teaching*					
Khogali	General cardiology	VLE	Yes	1 (positive)	Yes
Kaelber	General cardiology	VLE	Yes	1 (positive)	No
Gómez-Arbonés	General cardiology	VLE	Yes	1 (positive)	No
Bell	General cardiology	Self-Study Acceleration with Graphic Evidence (SAGE)	Yes	2 (positive)	Yes
Warriner	hypertension, cardiac failure, haemorrhagic shock	VLE	Yes	1 (positive)	Yes
Sacar	Peripheral arterial disease	VLE	Yes	1 (positive)	No
Raupach	Dyspnoea	VLE	Yes	2 (positive)	Yes
Pourmand	ECG teaching	Online learning module	Yes	2 (positive)	Yes
Zayed	Aorta-iliac disease, Carotid disease, DVT	Interactive learning module	Yes	1 (positive)	No
Weston	Heart Failure, Diabetes	Online seminars	Yes	3 (positive)	No
Maheshwari	Paediatric cardiology	E-learning	Yes	1 (positive)	No
McClusky	Cardiac anatomy	E-tutorial	Yes	1 (positive)	No
Dubner	General cardiology	Virtual symposium	No	None	No
Tuchinda	Cardiac auscultation	Online data base	Yes	None	No
Oliviera	Cardiac auscultation	Virtual interactive thorax	Yes	None	No
Sprick	Cardiac auscultation	Virtual patient interface	Yes	1 (positive)	No
Montassier	Cardiac auscultation	E-learning	Yes	2 (positive)	Yes
Petersson	Vascular anatomy	Quick time virtual reality movie	Yes	1 (negative)	No
Casillas	Cardiac rehabilitation	A website with commented slideshows	No	2 (negative)	No
*Computer based*					
Rothe	Cardiovascular knowledge	Interactive tutorials	Yes	1 (positive)	Yes
Karnath	Cardiovascular knowledge	Interactive heart model	Yes	2 (positive)	No
Butter	Cardiac auscultation	Computer tutorial	Yes	2 (positive)	No
Mahnke	Cardiac auscultation	Computer programme	Yes	2 (positive)	No
Criley	ECG	Computer programme	Yes	1 (positive)	No
*Mobile-Learning*					
Brewer	Cardiac interventions	Mobile simulator App	Yes	2 (positive)	Yes
Al-Jundi	Cardiovascular knowledge	MCQ App	Yes	1 (positive)	Yes
Torabi	Cardiovascular knowledge	Handbook App	Yes	1 (positive)	Yes
Bhathaji	Coronary interventions	App	Yes	1 (positive)	Yes
*Serious Gaming*					
De Sena	CPR	Video vs Serious gaming	Yes	2 (positive)	No
Coskun	Heart auscultation	Online game	Yes	2 (positive)	Yes
Drummond	Cardiac arrest training	Online course vs serious gaming	No	2 (negative)	No
*Podcasts*					
Sopka	BLS course	Podcast	No	2 (positive)	Yes
*Social Media*					
Liu	ECG teaching	Facebook, Twitter	Yes	2 (positive)	No
*Resourced-assisted self-directed learning (SDL)*					
Mahler	ECG teaching	SDL vs Lecture	No	2 (positive)	No
Raupach	ECG teaching	SDL vs Lecture	No	2 (positive)	No
Roppolo	Cardiovascular knowledge	SDL vs traditional teaching	No	2 (positive)	No
Pedersen	BLS course	MiniAnne kit	No	2 (positive)	No
Fuchs	Cardiac ultrasound	SDL	Yes	2 (positive)	No
Lam	Cardiac auscultation	Electronic stethoscope	Yes	2 (positive)	No
*Digital Resources*					
Camm	Cardiac auscultation	YouTube	Yes	1 (negative)	No
Azer	Cardiovascular mechanisms	YouTube, emedicine	Yes	1 (negative)	No
Azer	Cardiovascular disease	Wikipedia	Yes	1 (negative)	No
Results are tabulated as author; topic focus for teaching (topic); conceptual categories of teaching method delivery (method); whether the method was intended to supplement (yes) or replace (no) face-to-face teaching (supplement teaching); level of Kirkpatrick leaning outcome (Kirkpatrick Level) and whether Educational theory was discussed (Education Theory)					

### Online teaching

Twenty-two studies featured online access to cardiovascular learning resources. Here, learning was accessed and undertaken through the internet and intended to both support and replace face-to-face teaching. A popular method was a virtual learning environment (VLE) (n=6), a broad description for an online space to access teaching material though interactive text, photographs, videos, self-assessment and live-video sessions ( Khogali *et al*., 2011; Kaelber, Bierer and Carter, 2001; Gomez-Arbones *et al*., 2004; Warriner *et al*., 2017; Sacar *et al*., 2013; Raupach *et al*., 2010). This was used to deliver general cardiology themes through to specific topics such as hypertension, cardiac failure and peripheral arterial disease (PAD). Access to online learning did not deter student participation within timetabled teaching sessions; indeed it actively promoted engagement and participation ( Gomez-Arbones *et al.*, 2004).

Online modules, focused on specific subjects to gain knowledge and practical skills including vessel disease and ECG interpretation, were evaluated by four articles ( Pourmand *et al*., 2015; Montassier *et al*., 2018; Casillas and Gremeaux, 2012; Zayed, Lilo and Lee 2017). Compared to face-to-face teaching, e-learningdemonstrated non-inferiority compared with lecture-based learning ( Montassier *et al.,* 2015). Although participants found modules favourable, they identified a maximal learning session time of 90 minutes ( Casillas and Gremeaux, 2012).

Online seminars (live and recorded), tutorials and symposia presented as slides, webcasts, radio interviews and case presentations with self-assessment were used to deliver teaching multiple subjects ( Weston, Sciamanna and Nash, 2008; Dubner *et al.*, 2007; McCluskey *et al*, 2015; Maheshwari *et al.*, 2015). These were provided for post-graduate teaching and no evaluation was offered. A fourth article compared tutorials with a graphic add-on to printed guidelines for undergraduate teaching ( Bell *et al.*, 2000). Learning efficiency was improved, students required less time to study to achieve the same outcome in comparison to attending a tutorial and demonstrated greater learning satisfaction even over 6 months.

Online virtual patient interfaces were employed by three studies ( Tuchinda and Thompson, 2001; Oliveira *et al*., 2015; Sprick *et al*., 2008), useful for practical and knowledge skills, e.g. applying ECG pads and interpreting the results. Participant interaction was a key component and could be used within a group or self-learning session. A fourth article recreated vascular models from CT/MRI of prosections and compared them to an anatomy textbook to aid SDL however participants preferred dissection-based learning (Peteresson *et al*., 2008).

The majority of these studies (n=10, 53%) reported Kirkpatrick 1 learning outcomes although 3 reported no Kirkpatrick outcomes, summarised in Table 1. Five reported level 2 and one reported level 3 outcomes which demonstrated value in the content, accessibility and self-assessment. Five studies articulated eLearning theory which was appropriate for this method.

### Digital resources

Three articles focused on cardiovascular knowledge within the digital resources, Youtube, Wikipedia and eMedicine ( Camm, Sunderland and Camm, 2013; Azer, 2014; Azer *et al*., 2015) aimed to compliment face-to-face teaching. None reported Kirkpatrick outcomes or educational theory with a general lack of quality regulation and low academic accuracy and readability. Authors acknowledge Wikipedia is not intended to meet the needs of a medical audience. Although these resources allow a potential platform to disseminate learning resources for undergraduate teaching, they are in the public domain and susceptible to corruption by other users.

### Computer programs

Five articles focused on computer programs designed to provide teaching on specific subjects ( Rothe and Gersting, 2002; Karnath, Thornton and Das Carlo, 2003; Mahnke *et al*., 2004; Criley and Nelson, 2006; Butter *et al*., 2010). These included interactive tutorials, an animated heart, an interactive virtual patient simulator which allowed virtual auscultation and an interactive ECG analyser. These were intended for group-work and SDL. Kirkpatrick 2 outcomes were reported for 3 articles with outcome 1 with the remaining 2. Only one article articulated educational theory. The programs allowed repetitive and deliberate practice by participants to improve skills, particularly patient auscultation skills and could be a useful remediation tool for poorly achieving students ( Mahnke *et al*., 2004; Butter *et al*., 2010).

### Mobile-learning (M-learning)

Three articles presented mobile applications (apps), designed to be downloaded onto a handheld device and accessed on-the-go and one utilised mobile phones to deliver remote teaching and assessment ( Al-Jundi *et al*., 2017; Torabi, Khemka and Bateman, 2020; Bhatheja *et al*., 2018; Brewer *et al*., 2016). Apps included a cardiology handbook and two surgical training skills apps, designed as supplemental teaching to improve learning experiences. These reported that knowledge was gained in comparison to traditional reading although no understanding of accessibility and usability by their users was noted.

Kirkpatrick 1 learning outcomes were reported for 3 articles and level 2 outcome for one article. All articulated mobile learning theory. Ongoing assessment provided educators with real-time understanding of participant’s knowledge and resulted in a responsive and focused ongoing learning experience. Participants were satisfied with using this and engaged with the social aspects of teaching within this small group.

### Serious Gaming

Three articles focused on serious gaming, where a game is designed for a primary purpose other than entertainment and is designed to replace face-to-face teaching ( de Sena *et al*., 2020; Drummond *et al.*, 2017; Coskun, Adiguzel and Catak, 2019). All focused on practical skills - cardio-pulmonary resuscitation (CPR) and auscultation skills. For CPR training, skills gained through the serious game was equivocal to face-to-face and videos, although not statistically significant ( de Sena *et al*., 2020; Drummond *et al.*, 2017). For auscultation training, participants were exposed to repetitive heart sounds with knowledge retention to progress with the game. Although participants felt the ‘fun-factor’ was missing for this game, they continued to play as they were in a playful and competitive environment, which comply with medical students’ character ( de Sena *et al*., 2020). All reported Kirkpatrick 2 outcomes, although one applied articulated design and development research methodology ( Coskun, Adiguzel and Catak, 2019).

### Social media

These are websites and apps that users can use to share content and participate in a social network. One article used two sources, Facebook and Twitter, aiming to engage a virtual audience over a number of months for participant-only interaction ( Liu *et al*., 2017). A number of clinical vignettes were pre-agreed and ‘released’ to the audience weekly. The full capacity of the ‘social’ aspect of the network was not utilised, e.g. the vignettes were released without access to discussion or engagement with other users. This article demonstrated Kirkpatrick 1 outcome although did not articulate educational theory. Engagement decreased over the weeks. Those remaining at the end, were likely to be motivated learners and already had high scores pre-intervention with little knowledge improvement post-intervention.

### Self-directed learning (SDL) and resourced-assisted SDL

Six articles described SDL which may include methods described above ( Mahler *et al*., 2011; Roppolo *et al*., 2011; Pedersen *et al.*, 2018; Fuchs *et al*., 2018; Lam *et al*., 2004; Raupach *et al*., 2016). SDL was used to teach ECG interpretation and CPR training and compared these to face-to-face teaching methods which showed equivocal outcomes ( Mahler *et al*., 2011; Roppolo *et al*., 2011; Pedersen *et al*., 2018). Four articles utilised resources to complement the SDL and aimed to replace face-to-face teaching, which for CPR was an instructional DVD, blow-up manikin and cardboard training automated external deﬁbrillator ( Roppolo *et al*., 2011; Pedersen *et al*., 2018; Fuchs *et al*., 2018; Lam *et al*., 2004). Other SDL resources included a pocket cardiac ultrasound device and an electronic stethoscope both with the capacity to be used in isolation or with others. All demonstrated Kirkpatrick 2 outcomes, though no education theory. These allowed participants to practice cardiovascular skills under instruction, usually a video. These enabled greater learning exposure and practice and greater knowledge retention compared to instructor lead teaching, over time. When learning alone without the distraction of a group, participants may be more focused on the task, including higher self-monitoring resulting in favourable long-term effects of learning.

## Discussion

Cardiovascular teaching encompasses acquisition of knowledge and skills which has traditionally relied on contact teaching. Within this scoping review, we have presented 41 articles that described methods to teach cardiovascular knowledge or skills remotely which were intended for both self-directed and group work and have assessed learning outcomes using the Kirkpatrick framework.

These findings provide educators with an overview of the available literature and are transferable to other subjects. The relatively small number of articles is not surprising given this was previously a developing area of providing medical education. Although self-assessment was often incorporated in many studies, evaluation of individual teaching methods by each study was not always undertaken which is a missed opportunity. Clear factors which determine feasible transfer of teaching from contact into a remote forum have not been fully identified.

Most of the methods rely on technology, aligning with the adoption of the on-the-go learning embraced by the portability of smartphones and owned by more than 90% of the undergraduate population ( Gavali *et al*., 2017). They were intended to compliment or/and replace face-to-face teaching. Despite access to online learning, students have not been deterred from engaging with timetabled teaching sessions and remote learning can promote engagement with improved knowledge and confidence ( Gomez-Arbones *et al*., 2004). However the development of resources particularly M-learning/phone apps, is often costly and time-consuming meaning during the current pandemic medical schools seeking to adopt these methods would have to utilise existing platforms ( Chase, 2013).

Remote teaching methods are useful within multiple-environments, addressing teaching constraints including patient-exhaustion of multiple exams and lack of florid disease signs. These methods may allow participants within low and middle-income countries to access new teaching environments. However, articles only tested their method within a single cohort, timeframe and centre, thus does not address considerations such as quality assurance across cohorts, method longevity and how to accommodate content updates. Evaluation should also include feasibility, learner preference and cost-benefit e.g. learners may find a home resusucitation model beneficial but not feaible in many settings and incurs potentially substantial cost.

Our review associated VLEs, a popular method of decentralised learning, with cardiovascular knowledge improvement. A previous review identified that students already possess information technology and communication skills thus the ability to use online learning and social media effectively ( Ludmerer and Johns, 2005; Phungsuk, Viriyaveuakul and Ratanaolarn, 2017). Yet the ability to appropriately engage with remote SDL methods is not inherent and future research must be on how students can best utilise these resources for effective learning ( Agudo-Peregrina *et al*., 2014). However, the onus is on medical schools to ensure VLEs are learner-focused, have clear instruction and learning outcomes, and are easily navigated.

Investigation of social media, video-conferencing and online communication platforms such as Zoom and Google Teams, as methods of teaching are required which should exploit the far-reaching potential of such a media. The platforms may allow useful peer learning/small-group teaching particularly with the application of educational theory to guide its design, which demonstrates beneficial learning rather than a top-down teacher-learner tool ( Drummond and Delval, 2017). This has been seen within post-COVID-19 education with online small group configurations ( Rose, 2020). These tools could transcend geographic and program-specific boundaries ( Drummond *et al*., 2017).

Methods used for practical skills have reflected a practical component utilising virtual patients and serious gaming, which have largely resulted in a positive improvement. Virtual-reality and online-gaming are accessible and playable, hence popular and familiar. They create hype and motivation for further learning, especially if they have a fun-element ( Brewer *et al*., 2016). Having repetitive, one-to-one learning with feedback improved skills, confidence and satisfaction correlated with Gagne’s learning and the self-determination theory ( Coskun, Adiguzel and Catak, 2019). However, require specific equipment and software which reduces feasibility especially to a large learner cohort.

YouTube is currently the top three visited websites on the internet and has value within education as it is easily accessible even remotely, has relative ease to produce and upload content, and is complimented by free content. Users look to YouTube to seek information and entertainment. When evaluating CVD mechanisms on YouTube and Wikipedia, evaluation was not positive. However, as a platform, it may be an effective tool to enhance the learning experience if produced by educators for a specific purpose or as a complement to a learning session ( Moghavvemi *et al*., 2018).

Self-directed methods compared to face-to-face had mixed outcomes, aligning with previous findings ( Murad *et al*., 2010). Those which utilised specific resources were well received and improved cardiovascular knowledge/skills. These were designed for a specific skill or purpose with more defined instruction compared to SDL directed at a wider-focused subject. SDL is considered an important component of life-long learning and is a key competency in medical school curricula ( Eva *et al.*, 2004). However relevant skills for SDL should be taught to ensure participants can successfully engage with these activities rather than assuming they are inherent ( Gaines *et al*., 2018). Moreover, collaborating with other participants for peer support or utilising resources such as medical librarians may offer alternative avenues to gain these skills ( Gaines *et al.*, 2018; Leslie, 2017; Minuti *et al*., 2018).

Remote teaching does confer several challenges. Aside from the additional workload to convert some or all of the educational material into an appropriate remote method, requiring time and funding investment, there are logistical considerations including appropriate platforms to deliver teaching, adequate internet access and learner skills to engage with these tools. As future doctors, teaching aims to provide learners with the knowledge and skills to provide a compassionate, patient-centred medical service. Students and teachers alike thrive from the interaction and experience of face-to-face teaching, particularly authentic patient experiences, which is difficult to reproduce remotely.

## Review limitations

Firstly scoping literature for cardiovascular teaching may not be generalisable to other areas of the curriculum. As a broad review, it lacks focus on specific methods of remote learning. As only undergraduate studies were included, methods employed in other groups were not included. Finally, it is possible that we could have missed evidence of possible methods that may have never been reflected in the published or grey literature but are instead used in practice.

## Conclusion

The COVID-19 pandemic may be a watershed moment in medical education with the rapid, potentially permanent transition to remote provision of learning to compliment or substitute traditional teaching. However, significant gaps exist in the published literature regarding the feasibility, effectiveness and engagement with different remote learning methods. It is essential that medical educators conduct and report ongoing evaluations of remote teaching methods adopted during the COVID-19 pandemic to share experience and best-practice.

## Implications of the findings for research and practice

What should and can be done during the pandemic to fill research gaps including conducting remote student assessment/examination?

What should be undertaken once remote learning can be evaluated properly in comparison with more traditional methods of teaching?

What is the impact of remote learning on clinical performance?

What resources/equipment/skills are needed to deliver effective remote learning and can these be provided in low- and middle-income countries?

## Take Home Messages

• Remote teaching methods are available to compliment or replace face-to-face cardiovascular teaching if this is not available
• Medical educators may utilise a variety of methods including online, digital resources and teaching
• These methods may provide educators with options to reach medical students in both high, and low and middle income countries

## Notes On Contributors

**Dr Bernadeta Bridgwood** is a NIHR recognised Academic clinical Lecturer within the George Davies Research Team, Department of Cardiovascular Sciences, University of Leicester and a practising GP in Derbyshire. ORCiD: https://orcid.org/0000-0003-2710-585X

**Mr Cezar Sabbagh** is a Lower Limb Clinical and Research Fellow in Vascular Surgery within the George Davies Research Team, Department of Cardiovascular Sciences, University of Leicester. Mr Sabbagh is also an Honorary Registrar within Leicester Vascular Institute, University Hospitals Leicester

**Mr John Houghton** is a Clinical Research Fellow in Vascular Surgery within the George Davies Research Team, Department of Cardiovascular Sciences, University of Leicester. Mr Houghton is also a Specialist Trainee in Vascular Surgery within Health Education East Midlands.ORCiD: https://orcid.org/0000-0002-9159-5307

**Mr Andrew Nickinson** is a Clinical Research Fellow in Vascular Surgery within the George Davies Research Team, Department of Cardiovascular Sciences and University of Leicester. Mr Nickinson is also a Higher Trainee in Vascular Surgery within Health Education Wessex. ORCiD: https://orcid.org/0000-0003-0562-9580

**Coral Pepper** is a Clinical Librarian at University Hospitals of Leicester NHS Trust. She supports evidence-based care and research in the Cardiovascular, Genetics, Haematology and Oncology, Nephrology and Urology departments.

**Professor Rob Sayers** is the George Davies Chair of Vascular Surgery at the University of Leicester. He manages the George Davies Research team focused on improving the treatment of chronic leg ischaemia and diabetic foot ulceration. He was previously President of the Vascular Society of Great Britain and Ireland and Chair of the Vascular Clinical Reference Group (CRG) for NHSEI. ORCiD: https://orcid.org/0000-0002-6889-4659

## References

[ref1] Agudo-Peregrina Á. F. Iglesias-Pradas S. Conde-Gonzalez M. Á. and Hernndez-Garcia Á. (2014) Can we predict success from log data in VLEs? Classification of interactions for learning analytics and their relation with performance in VLE-supported F2F and online learning. Computers in Human Behavior. 31, pp.542–550. 10.1016/j.chb.2013.05.031

[ref2] Al-Jundi W. Kayssi A. Papia G. and Dueck A. (2017) Smart(phone) Learning Experience Among Vascular Trainees Using a Response System Application. Journal of Surgical Education. 74(4), pp.638–643. 10.1016/j.jsurg.2016.12.006 28130100

[ref3] Arksey H. and O’Malley L. (2005) Scoping studies: towards a methodological framework. International Journal of Social Research Methodology. 8(1), pp.19–32. 10.1080/1364557032000119616

[ref4] Azer S. (2014) Mechanisms in cardiovascular diseases: how useful are medical textbooks, eMedicine, and YouTube? Advanced Physiology Education. 38(2), pp.124–134. 10.1152/advan.00149.2013 PMC4056178 25039083

[ref5] Azer S. A. Alswaidan N. M. Alshwairikh L. A and AlShammari J. M. (2015) Accuracy and readability of cardiovascular entries on Wikipedia: are they reliable learning resources for medical students? BMJ open. 5(10), pp. e008187. 10.1136/bmjopen-2015-008187 PMC4606442 26443650

[ref6] Bell D. S. Fonarow G. C. Hays R. D. and Magnione C. M. (2000) Self-study from web-based and printed guideline materials. A randomized, controlled trial among resident physicians. Annals of Internal Medicine. 132(12), pp.938–946. 10.7326/0003-4819-132-12-200006200-00003 10858176

[ref7] Bhatheja S. Fuster V. Chamaria S. Kakkar S. (2018) Developing a Mobile Application for Global Cardiovascular Education. Journal of the American College of Cardiology. 72(20), pp.2518–2527. 10.1016/j.jacc.2018.08.2183 30442294

[ref8] Brewer Z. E. Ogden W. D. Fan J. I. Burdon T. A. (2016) Creation and Global Deployment of a Mobile, Application-Based Cognitive Simulator for Cardiac Surgical Procedures. Seminars in Thoracic and Cardiovascular Surgery. 28(1), pp.1–9. 10.1053/j.semtcvs.2016.02.006 27568126

[ref9] Butter J. McGaghie W. C. Cohen E. R. Kaye M. (2010) Simulation-based mastery learning improves cardiac auscultation skills in medical students. Journal of General Internal Medicine. 25(8), pp.780–785. 10.1007/s11606-010-1309-x 20339952 PMC2896602

[ref10] Caillas J. and Gremeaux V. (2012) Evaluation of medical students’ expectations for multimedia teaching materials: Illustration by an original method using the evaluation of a web site on cardiovascular rehabilitation. Annals of Physical and Rehabilitation Medicine. 55(1), pp.25–37. 10.1016/j.rehab.2011.12.001 22225845

[ref11] Camm C. F. Sunderland N. and Camm A. J. (2013) A quality assessment of cardiac auscultation material on YouTube. Clinical Cardiology. 36(2), pp.77–81. 10.1002/clc.22080 23172251 PMC6649466

[ref12] Cabral-Isabedra C. (2016) Google strikes deal with NHS that gives AI unit access to 1.6 million patient records, Tech Times. Available at: http://www.techtimes.com/articles/155059/20160501/google-strikes-deal-with-nhs-that-gives-ai-unit-access-to-1-6-million-patient-records.htm( Accessed: 17 May 2016).

[ref13] Chase J. (2013) Ipads and other drugs. Medical Mark Media.pp.10–11.

[ref14] Skochelak S. and Stack S. J. (2017) Creating the medical school of the future. Academic Medicine. 92(1), pp.16–19. 10.1097/ACM.0000000000001160 27008357

[ref15] Coskun Z. N. Adiguzel T. and Catak G. (2019) Acoustic Labyrinth: Validation of a Game-Based Heart Auscultation Educational Tool. World Journal on Educational Technology: Current Issues. 11(4), pp.245–256.

[ref16] Criley J. M. and Nelson W. P. (2006) Virtual tools for teaching electrocardiographic rhythm analysis. Journal of Electrocardiology. 39(1), pp.113–119. 10.1016/j.jelectrocard.2005.07.002 16387064

[ref17] De Sena D. P. Fabrício D. D da Silva V. D. Bodanese L. C. (2020) Comparative evaluation of video-based on-line course versus serious game for training medical students in cardiopulmonary resuscitation: A randomised trial. PLoS One. 14(4), pp. e0214722. 10.1371/journal.pone.0214722 PMC6453387 30958836

[ref18] Drummond D. Delval P. Abdenouri S. Truchot J. (2017) Serious game versus online course for pretraining medical students before a simulation-based mastery learning course on cardiopulmonary resuscitation: A randomised controlled study. European Journal of Anaesthesiology. 34(12), pp.836–844. 10.1097/EJA.0000000000000675 28731928

[ref19] Dubner S. J. Moss A. J. Schapachnik E. S. Levine P. A. (2007) Web-based virtual cardiac symposia: a new approach for worldwide professional medical education. Annals of Noninvasive Electrocardiology. 12(2), pp.165–170. 10.1111/j.1542-474X.2007.00156.x 17593186 PMC6932499

[ref20] Eva K. W. Cunnington J. P. Reiter J. I. Keane D. R. (2004) How can i know what i don’t know? Poor self assessment in a well-defined domain. Advanced Health Science Education. 9(3), pp.211–224. 10.1023/B:AHSE.0000038209.65714.d4 15316272

[ref21] Fuchs L. Gilad D. Mizrakli Y. Sadeh R. (2018) Self-learning of point-of-care cardiac ultrasound - Can medical students teach themselves? PLoS One. 13(9), pp. e0204087. 10.1371/journal.pone.0204087 30260977 PMC6160010

[ref22] Gaines J. Blake L. Kouame G. Davies K. (2018) Partnering to analyse selection of resources by medical students fo case-based small group learning: a collaboration between librarians and medical educators. Medical Reference Services Quarterly. 37(3), pp.249–265. 10.1080/02763869.2018.1477709 30239306

[ref23] Gavali M. Khismatrao D. Gavali Y. and Patil K. B. (2017) Smartphone, the New Learning Aid amongst Medical Students. Journal of Clinical Diagnostic Research. 11(5), pp.JC05–JC08. 10.7860/jcdr/2017/20948.9826 PMC5483706 28658804

[ref24] Goh P. and Sander J. (2020) A vision and use of technology in medical education after COVID-19 pandemic. MedEdPublish. 9(1), pp.49. 10.15694/mep.2020.000049.1 38058893 PMC10697445

[ref25] Gomez-Arbones X. Ferreita A. Pique M. Roca J. (2004) A cardiological web as an adjunct to medical teaching: prospective analysis. Medical Teacher. 26(2), pp.187–189. 10.1080/01421590310001653991 15203530

[ref26] Jayawickramarajah P. T. (2001) WFME task force on defining international standards in basic medical education. Medical Education. 35(5) pp.515. 10.1046/j.1365-2923.2001.0949b.x 11328525

[ref27] Kaelber D. C. Bierer B. S and Carter J. R. (2001) A Web-based clinical curriculum on the cardiac exam. Academic Medicine. 76(5), pp.548–549. 10.1097/00001888-200105000-00092 11346597

[ref28] Karnath B. M Thornton W. and Das Carlo M. (2003) Pilot study of a computer-based self-teaching system in cardiac auscultation. Medical Education. 37(11), pp.1048–1049. 10.1046/j.1365-2923.2003.01643.x 14629447

[ref29] Khogali S. E Davies D. A. Donnan P. T Gray A. (2011) Integration of e-learning resources into a medical school curriculum. Medical Teacher. 33(4), pp311–318. 10.3109/0142159X.2011.540270 21456989

[ref30] Lam C. S. Cheong P. Y. Ong B. K. and Ho K. Y. (2004) Teaching cardiac auscultation without patient contact. Medical Education. 38(11), pp1184–1185. 10.1111/j.1365-2929.2004.01989.x 15507015

[ref31] Leslie S. (2017) Designing an interactive web-based tutorial for health sciences students: a collaborative library project. Medical Reference Services Quarterly. 36(1), pp.90–96. 10.1080/02763869.2017.1259925 28112636

[ref32] Liu S. S. Zakaria S. Vaidya D. and Srivastava M. C. (2017) Electrocardiogram training for residents: A curriculum based on Facebook and Twitter. Journal of Electrocardiology. 50(5), pp.646–651. 10.1016/j.jelectrocard.2017.04.010 28479090

[ref33] Ludmerer K. M. and Johns M. M. (2005) Reforming Graduate Medical Education. JAMA. 294(9), pp.1083. 10.1001/jama.294.9.1083 16145029

[ref34] Maheshwari S. Zheleva B. Rajasekhar V. and Batra B. (2015) e-Teaching in pediatric cardiology: A paradigm shift. Annals of Pediatric Cardiology. 8(1), pp.10–13. 10.4103/0974-2069.149512 25684881 PMC4322394

[ref35] Mahler S. A. Wolcott C. J. Swoboda T, K. Wang H. (2011) Techniques for teaching electrocardiogram interpretation: self-directed learning is less effective than a workshop or lecture. Medical Education. 45(4), pp.347–353. 10.1111/j.1365-2923.2010.03891.x 21401682

[ref36] Mahnke C. B. Nowalk A. Hofkosh S. Zuberbuhler J. R. (2004) Comparison of two educational interventions on pediatric resident auscultation skills. Pediatrics. 113(5), pp.1331–1335. 10.1542/peds.113.5.1331 15121949

[ref37] McCluskey D. Woods J. Hashim M. Linn A. (2015) Developing an e-tutorial of heart anatomy with realtime 3d cadaveric prosections and cardiac imaging techniques. EDULEARN.pp2446–2455.

[ref38] Minuti A. Sorensen K. Schwartz R. King W. S. (2018) Libarians flip for students: teaching searching skills to medical students using a flipped classroom approach. Medical Reference Services Quarterly. 37(2), pp.119–131. 10.1080/02763869.2018.1439184 29558336

[ref39] Moghavvemi S. Sulaiman A. Jaafar N. and Kasem N (2018) Social media as a complementary learning tool for teaching and learning: The case of youtube. The International Journal of Management Education. 16(1), pp.37–42. 10.1016/j.ijme.2017.12.001

[ref40] Moher D. Liberati A. Tetzlaff J. and Altman D. for the PRISMA Group.(2009) Preferred Reporting Items for Systematic Reviews and MetaAnalyses: The PRISMA Statement. BMJ. 339pp.b2535. 10.1136/bmj.b2535 21603045 PMC3090117

[ref41] Montassier E. Hardouin J. Segard J. Batard E. (2015) e-Learning versus lecture-based courses in ECG interpretation for undergraduate medical students: a randomized noninferiority study. European Journal of Emergency Medicine: official journal of the European Society for Emergency Medicine. 23(2), pp.108–113. 10.1097/MEJ.0000000000000215 25386694

[ref42] Morris T. (2019) Self-directed learning: A fundamental competence in a rapidly changing world. International Revie of education. 65(4), pp.63–65. 10.1007/s11159-019-09793-2

[ref43] Murad M. Coto-Yglesias I. Varkey P. Prokop L. (2010) The effectiveness of self-directed learning in health professions education: a systematic review. Medical Education. 44(11), pp.1057–1068. 10.1111/j.1365-2923.2010.03750.x 20946476

[ref44] Nickinson A. T. Carey F. Tan K. Ali T (2020) Has the COVID-19 pandemic opened our eyes on the potential of digital teaching? A survey of UK vascular surgery and interventional radiology trainees. European Journal of Vascular and Endovascular Surgery. 60(6), pp.952–953. 10.1016/j.ejvs.2020.09.010 33129681 PMC7510498

[ref45] Oliveira I. Reis Z. Araújo M. and Freire C. (2015) Cardiac auscultation simulator embedded in virtual learning environment to support medical teaching. Studies in Health Technology and Informatics. 216pp.976. 26262278

[ref46] Pedersen T. H. Kasper N. Roman H. Egloff M. (2018) Self-learning basic life support: A randomised controlled trial on learning conditions. Resuscitation. 126, pp.147–153. 10.1016/j.resuscitation.2018.02.031 29522830

[ref47] Peters M. Godfrey C. McInereney P. Munn Z. . (2020) Chapter 1: Scoping Reviews (2020 version).In: Aromataris E Munn Z (Editors). JBI Manual for Evidence Synthesis, JBI. Available at: https://synthesismanual.jbi.global( Accessed 17 July 2020).

[ref48] Peteresson H. Sinkvist D. Wang C. and Smedby O. (2009) Web-Based Interactive 3D Visualization as a Tool for Improved Anatomy Learning. Anatomical Sciencs Education. 2(2), pp.61–68. 10.1002/ase.76 19363804

[ref49] Phungsuk R., P. Viryavejakul C. and Ratanaolarn T. (2017) Development of a problem-based learning model via a virtual learning environment. Kasetsart Journal of Social Sciences. 3, pp.297–306. 10.1016/j.kjss.2017.01.001

[ref50] Pourmand A. Tanski M. Davis S. Shokoohi H. (2015) Educational Technology Improves ECG Interpretation of Acute Myocardial Infarction among Medical Students and Emergency Medicine Residents. West Journal of Emergency Medicine: Integrating Emergency Care with Population Health. 16(1), pp.133–137. 10.5811/westjem.2014.12.23706 PMC4307697 25671022

[ref51] Raupach T. Harendza S. Anders S. Schuelper N. (2016) How can we improve teaching of ECG interpretation skills? Findings from a prospective randomised trial. Journal of Electrocardiology. 49(1), pp.7–12. 10.1016/j.jelectrocard.2015.10.004 26615874

[ref52] Raupach T. Munscher C. Pukrop T. Anders S. (2010) Significant increase in factual knowledge with web-assisted problem-based learning as part of an undergraduate cardio-respiratory curriculum. Advanced Health Science Education Theory Practice. 15(3), pp.349–356. 10.1007/s10459-009-9201-3 PMC2940026 19774475

[ref53] Roppolo L. P. Heymann R. Pepe P. and Wagner J. (2011) A randomized controlled trial comparing traditional training in cardiopulmonary resuscitation (CPR) to self-directed CPR learning in first year medical students: The two-person CPR study. Resuscitation. 82(3), pp.319–325. 10.1016/j.resuscitation.2010.10.025 21146914

[ref54] Rose S. (2020) Medical Student Education in the Time of COVID-19. JAMA. 323(21), pp.2131–2132. 10.1001/jama.2020.5227 32232420

[ref55] Rothe C. and Gersting J. (2002) Cardiovascular interactions: an interactive tutorial and mathematical model. Advances in Physiology Education. 26(1-4), pp98–109. 10.1152/advan.00031.2001 12031942

[ref56] Sacar M. Önem A. Büke S. and Baltalarlı A. (2013) The effect of distance-based learning on the fifth stage medical students’ perception in peripheral vascular diseases course: a questionnaire survey. Anadolu kardiyoloji dergisi: Anatolian J Cardiology. 13(3), pp275–277. 10.5152/akd.2013.079 23443855

[ref57] Sprick C. Ruthenbeck G. Owen H. and Reynolds K. (2008) Virtual patient monitors for new user familiarization. Studies in Health Technology & Informatics. 132, pp.484–486. 18391350

[ref58] The Cochrane Collaboration . (2021) Developmental, psychosocial and Learning Problems. Available at: https://dplp.cochrane.org/data-extraction-forms( Accessed: 10 July 2020).

[ref59] Torabi A. Khemka A. and Bateman P. (2020) A Cardiology Handbook App to Improve Medical Education for Internal Medicine Residents: Development and Usability Study. JMIR medical education. 6(1), pp. e14983. 10.2196/14983 32297866 PMC7193443

[ref60] Tuchinda C. and Thompson W. (2001) Cardiac auscultatory recording database: delivering heart sounds through the Internet. Proceedings. AMIA Symposium.pp.716–720. PMID: 11825279 11825279 PMC2243700

[ref61] Warriner D. R. Bayley M. Shi Y. Lawford P. V. (2017) Computer model for the cardiovascular system: development of an e-learning tool for teaching of medical students. BMC Medical Education. 17(1), pp.220. 10.1186/s12909-017-1058-1 29157229 PMC5697416

[ref62] Weston C. M. Sciamanna C. N. Nash D. B. (2008) Evaluating online continuing medical education seminars: evidence for improving clinical practices. American Journal of Medical Quality. 23(6), pp.475–483. 10.1177/1062860608325266 19001103

[ref63] World Health Organisation . (2021) Cardiovascular diseases. Available at: https://www.who.int/news-room/fact-sheets/detail/cardiovascular-diseases-(cvds)( Accessed: 11 July 2020).

[ref64] Zayed M. A. Lilo E. A. and Lee J. T. (2017) Impact of an Interactive Vascular Surgery Web-Based Educational Curriculum on Surgical Trainee Knowledge and Interest. Journal of Surgical Education. 74(2), pp. 251257. 10.1016/j.jsurg.2016.09.003 27727138

