# Supplementary material for: Medical Education in a Post COVID-19 era – remote teaching methods for cardiovascular knowledge and skills
Source: MedEdPublish (2016). 2021 Mar 3;10:62. [Version 1] doi: 10.15694/mep.2021.000062.1 (PMC10939632; doi:10.15694/mep.2021.000062.1)
Supplement: Peer review history

# Reviewer report: Reviewer response for version 1

**DOI:** 10.21956/mep.18879.r26945

**Published:** 2021-06-08

**Authors:** Ken Masters

**Affiliations:** 1 Sultan Qaboos University

This review has been migrated. The reviewer awarded 4 stars out of 5 A useful scoping literature review on remote teaching methods for cardiovascular knowledge and skills.The authors clearly lay out the context in which the advent of Covid-19 has led to a need for online cardiovascular teaching, and the scoping review sets out to determine the range and value of material available to medical educators. The study has been well-conducted, and Table 1 and the accompanying descriptive analysis gives a useful overview of the relevant material found.Two small issues I would like to see addressed:• Figure 1 should give some details of the 882 records removed in the screening process.• Table 1 should give the date of publication so that readers can have some idea of the chronological scope of the sources.Overall, however, a very useful read for understanding the range of literature currently available for online cardiovascular teaching and learning.

Reviewer Expertise:

NA

No decision status is available

# Reviewer report: Reviewer response for version 1

**DOI:** 10.21956/mep.18879.r26944

**Published:** 2021-03-09

**Authors:** Deb Halder

This review has been migrated. The reviewer awarded 4 stars out of 5 The paper has drawn thorough planning of how it tends to proceed to the online medical education owing to the experiences of incapacity from any party involved in acquiring and providing medical education.The paper needs to explain the limitation of on-line process of learning and teaching where blended learning has been thought the suitable way of dissemination education. Challenges are there in every type of method. But the arrangement of the paper how it evaluates the idea is satisfactory enough.

Reviewer Expertise:

NA

No decision status is available

# Reviewer report: Reviewer response for version 1

**DOI:** 10.21956/mep.18879.r26946

**Published:** 2021-03-03

**Authors:** Subha Ramani

**Affiliations:** 1 Harvard Medical School, Brigham and Women's Hospital

This review has been migrated. The reviewer awarded 4 stars out of 5 Being someone whose clinical skills training was entirely face to face, i found this article interesting to read. The authors have done a very good job in their scoping review and provided a variety of options for virtual teaching of cardiovascular examination skills. The covid pandemic has necessitated these creative approaches. The options described can be applied to the teaching of clinical exam of other organ systems.The paper is well written and well referenced.The methods listed could supplement in-person teaching and exam post pandemic.I have a couple of comments for reflection:1. How can live teaching build on the multimedia strategies. Eventually, we all need to get accustomed to in-person exam and all clinicians need these vital skills. Some things just cannot be done virtually in the clinical environment2. We have a list of virtual strategies, we do not know the impact of each of these and need robust program evaluation to know what works and what does not.This article will be useful to all clinical teachers engaged in teaching clinical skills and wish to supplement virtual strategies to live teaching.

Reviewer Expertise:

NA

No decision status is available
